# A roadmap to reduce information inequities in disability with digital health and natural language processing

**DOI:** 10.1371/journal.pdig.0000135

**Published:** 2022-11-17

**Authors:** Denis R. Newman-Griffis, Max B. Hurwitz, Gina P. McKernan, Amy J. Houtrow, Brad E. Dicianno

**Affiliations:** 1 Department of Biomedical Informatics, University of Pittsburgh, Pittsburgh, Pennsylvania, United States of America; 2 Center for Health Equity Research and Promotion, VA Pittsburgh Healthcare System, Pittsburgh, Pennsylvania, United States of America; 3 Information School, University of Sheffield, Sheffield, United Kingdom; 4 Department of Physical Medicine and Rehabilitation, University of Pittsburgh, Pittsburgh, Pennsylvania, United States of America; 5 Human Engineering Research Laboratories, VA Pittsburgh Healthcare System, Pittsburgh, Pennsylvania, United States of America; Massachusetts General Hospital, UNITED STATES

## Abstract

People with disabilities disproportionately experience negative health outcomes. Purposeful analysis of information on all aspects of the experience of disability across individuals and populations can guide interventions to reduce health inequities in care and outcomes. Such an analysis requires more holistic information on individual function, precursors and predictors, and environmental and personal factors than is systematically collected in current practice. We identify 3 key information barriers to more equitable information: (1) a lack of information on contextual factors that affect a person’s experience of function; (2) underemphasis of the patient’s voice, perspective, and goals in the electronic health record; and (3) a lack of standardized locations in the electronic health record to record observations of function and context. Through analysis of rehabilitation data, we have identified ways to mitigate these barriers through the development of digital health technologies to better capture and analyze information about the experience of function. We propose 3 directions for future research on using digital health technologies, particularly natural language processing (NLP), to facilitate capturing a more holistic picture of a patient’s unique experience: (1) analyzing existing information on function in free text documentation; (2) developing new NLP-driven methods to collect information on contextual factors; and (3) collecting and analyzing patient-reported descriptions of personal perceptions and goals. Multidisciplinary collaboration between rehabilitation experts and data scientists to advance these research directions will yield practical technologies to help reduce inequities and improve care for all populations.

## Author summary

As medical successes have increased lifespans and improved health outcomes around the world, global ageing and more frequent occurrence of chronic diseases are contributing to a corresponding increase in the impact of disability worldwide. People with disabilities often experience injustices and ableism in healthcare, and as the prevalence and impacts of disability continue to grow, there is a clear need for more disability-focused and disability-aware practices in healthcare. Here, we present a roadmap towards bringing better information on the concrete impacts and lived experience of disability into healthcare practice, as a key step towards addressing this need. We describe 3 major barriers to collecting and analyzing information on people’s experience of function and disability, including a lack of contextual information, underemphasis of the patient’s voice and experience, and technical challenges in recording disability-related information in health records. These barriers create significant information inequities that contribute to the disparities in health and healthcare experienced by disabled people. We further propose 3 specific directions for new research and development on using digital health technologies, particularly natural language processing (NLP) and other artificial intelligence technologies, to help mitigate these barriers and support more equitable healthcare for people with disabilities.

## Introduction

Disability affects over 25% of adults in the United States, and the number of people experiencing disability globally is rapidly increasing [1]. People with disabilities experience disparities in health and healthcare, from higher hospital readmission rates to greater multimorbidity and mortality risk [2–4] and are faced with reduced access to care and lower quality of care received [5,6]. While disparities disproportionately affect disabled people, the disparate outcomes themselves are not caused solely by disability and are largely avoidable [7,8]. The International Classification of Functioning, Disability and Health (ICF)—the international standard conceptual model of function and disability—represents disability as a multidimensional phenomenon deeply entwined with a person’s health and with barriers and facilitators in their environments [9]. This framework thus provides an invaluable lens for understanding how disability and disparities interact, and how disparities are magnified when disability intersects with other marginalized identities and combinations of ableism, racism, and gender discrimination reduce opportunities for employment, housing, education, and community activity [10,11].

This multidimensional lens presents 2 key questions for reducing disparities for people with disabilities: understanding where the disparities come from (i.e., contributions of environmental and personal factors in addition to health condition and impairment) and how they are realized in peoples’ lived experiences. Many factors affect disparities in outcomes, from bias in provider interactions [12,13] and quality of services received [14,15] to structural disparities in access to healthcare [16,17] and rates of poverty [18,19]. By knowing what to look for, we can identify people at risk early and tailor the services they receive to support their underlying needs [8,20]. Each person may also experience impairments or functional limitations in different ways; for example, reduced mobility can affect getting to work, meeting with family and friends, and other activities to varying degrees. By understanding how a person’s functioning affects their experience of their own health and their priorities, we can better address what matters most to them and achieve person-centered goals in their care.

**Fig 1:**
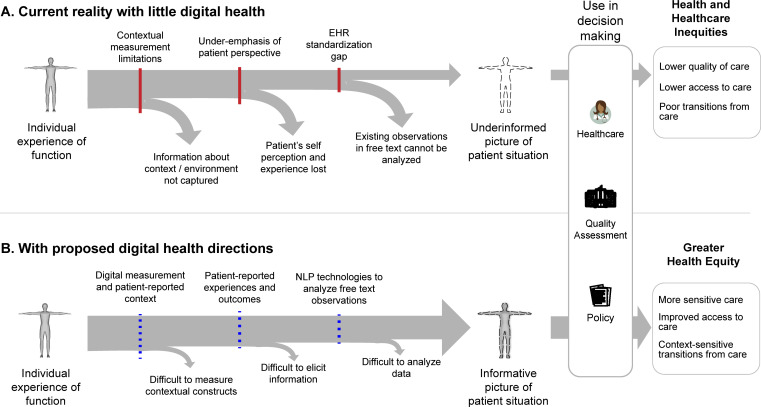
Information barriers to a complete picture of patients’ unique situations in EHR create information inequities at multiple levels. Panel (A): We describe 3 significant information barriers in current practice, which significantly reduce the amount of information about patients’ situations recorded in the EHR and negatively impact decisions made about patient care based on the EHR. Panel (B): We identify 3 directions for new research on digital health technologies to facilitate better understanding patient experience. EHR, electronic health record.

In this narrative review, we draw on an interdisciplinary set of references across the digital health, artificial intelligence, physical medicine and rehabilitation, and disability studies literatures to illustrate current challenges to understanding people’s unique situations in healthcare and identify opportunities for addressing these challenges with digital health technologies. We focus on information about a person’s situation captured during clinical encounters as the primary source of information used in healthcare decisions. The electronic health record (EHR) helps to aggregate and communicate health information for providing optimal care, and thus presents significant potential for understanding the origin and experience of disparities in the experience of function [21]. However, the practical value of EHR data is often limited by barriers of technology and practice that restrict our ability to document and analyze information on individual needs, priorities, and experiences [22]. This lack of individualized information creates *information inequities* that contribute to inequitable processes and outcomes across patient care, health system administration, and social support programs. As illustrated in Fig 1, we define 3 key information barriers that lead to loss of valuable information and understanding about peoples’ unique situations in their roles as patients, directly contributing to these inequities. We further describe 3 ways in which digital health technologies—particularly natural language processing (NLP)—can help capture more of this important information and facilitate multidimensional understanding of patient needs.

The language used to discuss disability is an important factor in the inclusion or marginalization of disabled people [23,24]. In this article, we use a mixture of the identity-focused “disabled people,” to reflect the nature of disability as an identity and political category; the person-focused “people with disabilities,” to reflect a person-first approach; and “persons experiencing disability,” to reflect the conceptualization (reified in the ICF) of disability as a *phenomenon* produced in the interaction of a person with their environment. We refer to “patients’ situations” and “patient needs” to reflect our focus on digital health technologies specifically in the healthcare setting, in which people assume a patient role.

### Multidimensional factors of disability

**Fig 2:**
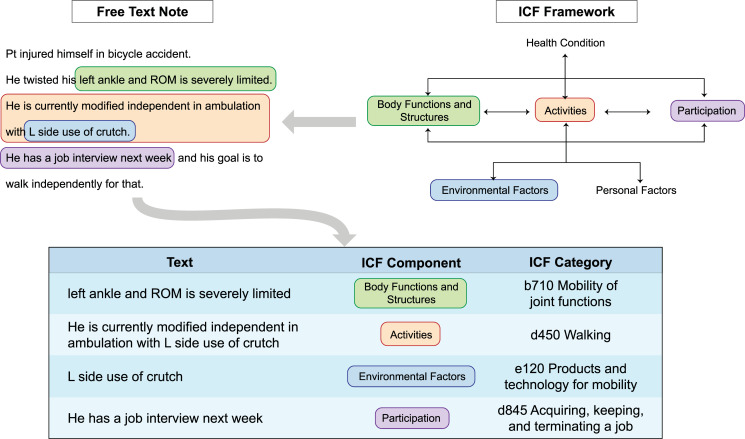
Examples of information related to different domains of the ICF in free text data. Diverse data, including information in free text, can be linked to standardized categories in the ICF classification, providing an internationally recognized conceptual structure for capturing multidimensional factors of patients’ functional experience. NLP technologies for information extraction and normalization can serve to perform this linking automatically, making free text observations accessible for analysis and insight. ICF, International Classification of Functioning, Disability and Health; NLP, natural language processing.

Clearly defining the factors affecting disability is the first step for effective analysis. We draw on the common language of the ICF, which presents a biopsychosocial model defining disability as “a multidimensional phenomenon resulting from the interaction between people and their physical and social environment,” including impairments in body functions and structures, activity limitations, and participation restrictions [9]. The ICF framework enables capturing a deep understanding of the multiple factors affecting an individual’s experience, including both health characteristics and contextual factors. Fig 2 illustrates how the ICF can be used to ground analysis of valuable observations in EHR, mapping information about impairments to body functions or structures, limitations in functional activity, environmental factors such as use of assistive devices, and social participation and priorities to standardized categories within the ICF framework. The ICF thus provides a powerful multidimensional framework to represent patients’ unique situations and help guide more equitable decision-making.

### Information barriers to a multidimensional picture of disability in practice

**Fig 3:**
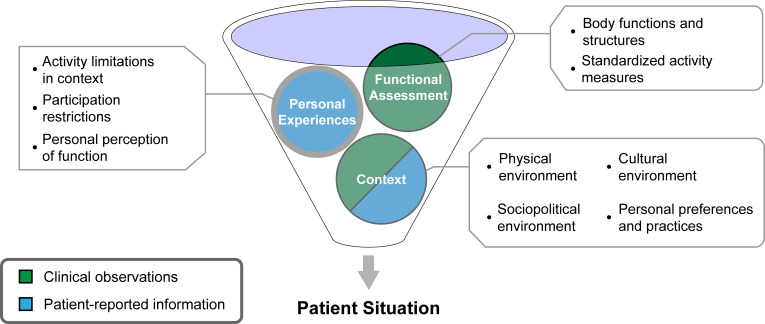
Conceptual illustration of the multiple dimensions of health data needed to fill in the picture of a patient’s functional situation. Capturing these data requires information both from clinical observations and patient-provided information. Without including all of functional assessments, the patient’s personal experiences, and their context, the overall picture of the patient’s situation is incomplete.

Using individual-level data such as that within the EHR to inform efforts to address disparities in the experience of disability faces significant practical and technical barriers. Translating health data into a multidimensional picture of an individual’s experience requires synthesizing information on function (including body functions, activities, and participation), personal experience (including behaviors and choices), and contextual factors that may reflect structural or individual inequities. This requires both clinical observations—such as in histories, physical examinations, and activity measures—and patient-provided information. As Fig 3 illustrates, these sources of information may require multiple means of assessment and data collection, and often complement one another by capturing different aspects of a patient’s situation.

We highlight 3 major barriers in current practice to recording and analyzing this information: (1) the challenge of measuring a patient’s contexts of functioning; (2) underemphasis of the patient’s perspective (affecting both personal experiences and context); and (3) the technological gap in EHRs (limiting the availability and utility of function, personal experience, and context information). The interaction of these barriers reduces the information available in the EHR about the individual experience of disability and about key personal and environmental factors that help guide treatment plans and identify needs.

#### Barrier 1: The contextual measurement challenge

Functional outcomes are not determined by an individual’s health state alone, but result from the interaction of an individual with their physical, social, and cultural environment. Thus, any picture of a person’s function is incomplete while it lacks information on the contexts they live in, which the ICF separates into personal and environmental factors. Contextual factors have been shown to be critical for measuring both overall function [25,26] and work outcomes [27].

However, measuring contextual factors in a standardized way remains a significant challenge [28]. While some functional assessment instruments have been developed to include measurement of contextual factors [29], such measures are burdensome to employ in regular clinical practice, and the lack of standardization around contextual factors precludes their structured documentation for easy use. Integration of contextual measurement in EHRs is minimal, limited by the cost of customization and lack of connectivity between EHRs and other technologies well-suited for capturing information on context, such as mobile devices and passive sensors [30]. Development of initial frameworks for capturing social determinants of health (SDOH), which capture both personal and environmental factors [31], illustrate a potential path forward leveraging both structured and unstructured data [32], though their use has been complicated by the lack of shared standards for what to document and how [33]. Broader documentation of contextual factors will require surmounting similar challenges.

#### Barrier 2: Underemphasis of the patient’s perspective

Under the medical model of disability, individuals experiencing disability have historically been reduced to a diagnosis of physical, cognitive, and emotional impairments, and healthcare has focused on providing treatments to fix a “problem.” This way of thinking medicalizes the experience of disability and embeds ableism into the structure of health systems and the fabric of the healthcare process. Even healthcare providers with specialized experience and training to work with people with disabilities can exhibit a biased view of disability as inherently negative [34], and people with a disability consistently rate their quality of life higher than others perceive it [35]. Additionally, providers often dismiss and remain ignorant of different kinds of disability experiences, and in an effort to fix perceived “problems,” may miss the mark on patient-centered care and patient-driven goals.

This medicalization of disability both contributes to and is exacerbated by the underemphasis of the patient’s perspective—i.e., the patient’s views on their own health and functioning and their priorities for care and quality of life—in the health record. Epistemic injustice is often encouraged by privileging medical assessment over personal experience, elevating the clinician’s status and diminishing the patient’s perspective [36]. At the same time, medical professionals may disregard or deem unreliable information reported by disabled people [37]. While clinical expertise is undeniably important, overvaluing one’s own expertise can lead to medical errors [38], and a medical perspective of disability as a pathology needing treatment can ignore personal values of community and culture [39]. This affects the doctor–patient relationship, and persons experiencing disability often feel ignored and left out of treatment decisions [40]. As Peña-Guzmán [38] argues, “Medical information is wholly insufficient to understand the lived experience of a person with a particular impairment, an experience saturated with social, cultural, political, and historical complexities typically untouched by even the best and most capacious forms of medical education.”

Collecting information on the patient’s perspective and experience is rarely practiced in patient encounters. This information is thus largely missing from health documentation and unavailable to inform clinical decision-making. The increasing adoption of patient portals and technologies for collecting patient-reported information outside the clinic (such as mobile health (mHealth)) are valuable steps towards providing patients with the ability to communicate to their providers what is important to them and what is affecting their daily lives. Such tools to help bring the patient’s perspective into the clinical conversation are key to advancing shared decision-making for patient-centered care [41,42].

#### Barrier 3: The EHR standardization gap

The inherent tension in EHRs between standardization and expressivity is a core challenge of recording and using multidimensional information on patients’ unique experience of function and disability [43]. Standardization makes communicating and utilizing information easier and enhances EHR data sharing and reuse in research and practice [21,44]. Some rehabilitation disciplines have been making initial strides in standardizing outcome reporting [45,46], and the ICF has been explored as a practical coding system [47,48]. However, the lack of implementation standards for capturing data on function or context in EHR systems—and the conceptual difficulty of mapping unique factors of context and personal experience to clearly defined data fields—often leaves standardization out of reach in practice [22]. Function, personal experience, and context are thus primarily documented in free text fields in the EHR [49,50]. This imposes burdens on clinicians, requiring time-consuming documentation that may conflict with administrative and reimbursement demands [51] as well as searching through narrative records for information that varies in consistency [22], and limits the ability of health systems to leverage this information in administrative decision-making [52].

**Fig 4:**
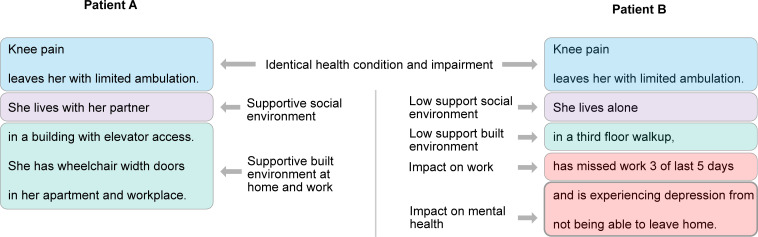
Example of differences in environmental factors for patients with identical health conditions and impairments. Both patients would score the same on unassisted walk tests, but the additional information provided distinguishes between a patient with access to facilitators in relevant environments (Patient A) and one facing significant environmental barriers (Patient B). The text highlighting indicates conceptual organization of information only; however, a similar analysis could be conducted automatically with NLP techniques. NLP, natural language processing.

However, free text documentation also has significant advantages for capturing information on function, personal experience, and context. The expressivity of natural language allows both clinicians and patients to describe complex interactions between people and their environment and add salient details beyond what standardized measures of function can capture [22]. This expressivity is key for distinguishing between different individuals’ needs for care, such as their own perception of their functioning or the contextual factors that differentiate between an inconvenience and a severe limitation. As Fig 4 illustrates, the same health condition and/or impairment may have minimal impact on function in a supportive environment, but lead to significant impacts on function and well-being in non-supportive environments. These highly individualized differences are difficult to capture in standardized data fields, but natural to communicate in a free text description. Thus, moving towards more value-based care that adapts to the needs of diverse individuals requires balancing standardization and the time and effort required in healthcare documentation with documentation that is expressive enough to record the complex factors contributing to a patient’s unique experience.

### Digital health facilitators: How better technologies can help efforts to advance equity

Advances in medical data science and AI technologies offer new opportunities to help mitigate these barriers—and reduce the information inequities they create—by making it easier to record and analyze a more multidimensional picture of individual function. As we redefine and adapt data collection practices to support the growing need for rehabilitation care [1], we can focus on combining structured data elements such as performance measures and other standardized information on functional outcomes with the flexibility and expressivity of free text documentation to collect a more nuanced picture of individual context and personal experience [53].

**Fig 5:**
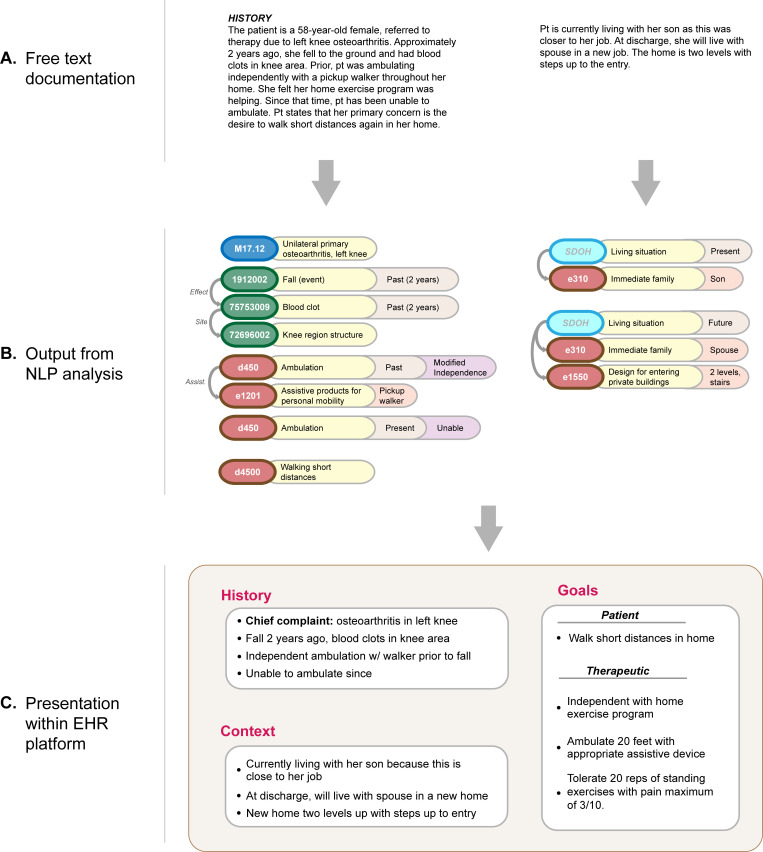
Conceptual illustration of the use of NLP to analyze and organize complex information recorded in free text clinical notes. Panel (A): Two example free text notes with important information about a patient’s situation. Panel (B): Example output of an NLP system designed to extract and standardize pieces of information described in text, such as medical events, diagnoses and symptoms, functional outcomes, and contextual factors. The individual steps of the NLP pipeline are omitted for brevity. Panel (C): This information can then be used for a variety of purposes, from recommendation of therapeutic interventions to organizing key information into an EHR dashboard for quick review in patient care (as illustrated in the mock dashboard here). EHR, electronic health record; NLP, natural language processing.

Within the range of digital health technologies, NLP holds particular promise for enabling more equitable data capture and analysis on factors of function. NLP technologies enable analysis and standardization of free text data while retaining the human detail and ease of use of natural language. Fig 5 illustrates a conceptual example of NLP analysis. In this example, the free text is first analyzed to identify where individual pieces of medical and functional information are mentioned. These spans of text are then mapped to standardized categories in controlled vocabularies (e.g., ICD-9 codes, ICF categories, SNOMED CT codes) and analyzed to identify other relevant attributes such as historicity or details within a standardized category (e.g., “spouse” as a specific member of immediate family). Individual pieces of information are then linked to one another to reflect their relationships: e.g., that a fall led to a blood clot located in the knee. Finally, this structured information is synthesized into natural language descriptions and displayed to the provider in the EHR platform. This example does not reflect a specific existing NLP pipeline, but rather serves to illustrate a variety of relevant analyses that can be conducted using NLP technologies.

**Fig 6:**
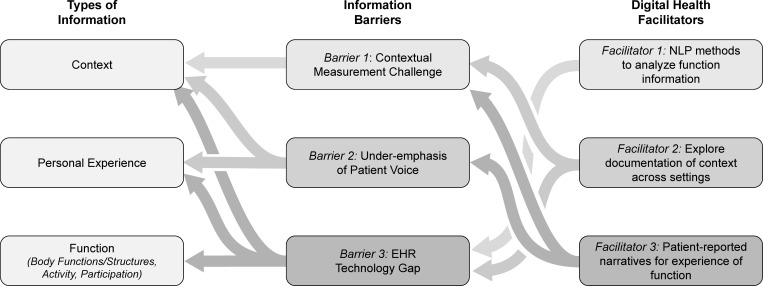
Relationships between multidimensional information on a patient’s situation information barriers limiting collection of this information and ways in which digital health technologies such as NLP can serve as facilitators to lower these barriers. NLP, natural language processing.

We describe 3 directions for future research on using digital health technologies as facilitators to help address the 3 barriers we have identified, with a particular focus on NLP tools and methodologies. These research directions can be realized through multidisciplinary collaborations between rehabilitation experts and experts in AI and data science. The relationships between multidimensional patient information, the information barriers we identify, and NLP-based facilitators are illustrated in Fig 6. Our 3 facilitators are described in order of increasing scope, beginning with technologies achievable in the short term with the use of existing EHR data from rehabilitation and other health care.

#### Facilitator 1: Development of NLP technologies to analyze information on functioning in EHR notes

Rich information on individual functional status is already frequently recorded in free text notes in the medical record [49,54]. Studies of the language used by providers and patients to describe functional outcomes [55,56] and analyses of the linguistic structure of these descriptions [57,58] have laid valuable groundwork for developing NLP systems attuned to the language of function.

Technological solutions to extract and organize this information (including descriptions of impairments, activity limitations, and participation restrictions) from free text are the focus of a growing body of research. Kukafka and colleagues [59] reported one of the first studies on coding EHR free text for patient functioning, targeting 5 distinct activity types. Agaronnik and colleagues [50,60] used NLP to extract data about wheelchair use from EHR narratives and to identify the frequency with which functioning was documented in oncology notes. Newman-Griffis and colleagues developed methods for extracting information about limitations in daily living activities from EHR text, including linkage to the ICF [61–63]. Functioning information has also been investigated in targeted contexts such as geriatric syndrome [64,65] and frailty [66].

Translating such technologies to practical settings requires new cross-disciplinary research by rehabilitation scientists and clinicians together with experts in NLP and AI, to investigate the range of real-world information needs in rehabilitation settings and develop use-grounded standards for evaluating NLP technologies in practice. Advancing research in this area will empower analysis of the rich information already recorded in existing clinical rehabilitation records while maintaining the detail and flexibility of free text clinical narratives.

#### Facilitator 2: Exploration of contextual documentation across encounter settings

Documenting environmental and personal factors affecting a person’s function is an important part of identifying interventions and environmental modifications to maintain or restore function [67,68]. The expressivity of free text allows for salient aspects of a person’s context to be documented in human detail, without restricting it to standardized categories that may not capture relevant aspects of the environment or person [28,69]. While the contextual measurement challenge limits the availability of structured data on context from standardized instruments, significant information on patient environment and personal factors is often recorded in free text notes from clinical encounters. This information is not yet well characterized, and NLP-driven analysis of notes combined with analysis of relevant billing codes and/or standardized measures where available can provide valuable insight into what kinds of contextual factors are documented in current practice, when, and in what form.

In addition, encounters in different clinical settings lend themselves to documenting different types of information. For example, an analysis of notes from physical therapy encounters for inpatient stroke rehabilitation found some information on environmental factors [54]. Other settings such as outpatient and transitional rehabilitation, as well as telehealth consultations and social work encounters, are also well positioned for providers to record key details of a patient’s physical and social environment. Analyzing documentation across various clinical settings will help to identify future opportunities presented by shifts in care delivery methods (such as increased use of telehealth) and documentation practice for capturing greater detail on individuals’ context.

#### Facilitator 3: Patient-reported narratives of functioning and disability

The patient themselves is the best source for learning about their own perspective on their functioning and their priorities and personal goals in their healthcare. Patient-reported outcome measures (PROs) have experienced rapid growth in research focus and clinical application in recent years [70,71]. PRO usage has been growing in practice, though numerous logistical, administrative, and patient challenges remain limiting factors for broader uptake [72,73]. Newer measures are allowing patients to provide broader information on their functioning, enabling recording of functional status outside of the clinical setting [74].

The potential for free text items in PROs, with which patients can freely describe salient aspects of their functional experience, has yet to be explored. As methods to integrate PROs into EHR systems continue to develop [75], narrative items offer a potential route to combine the patient’s perspective and experience together with clinical observations in the EHR. The OpenNotes effort has demonstrated the potential of this approach in access to the record [76]; similar linkage to PRO instruments can further amplify personal experience. Eliciting patient perspectives and preferences poses significant challenges, but the potential impact in patient-centered decision-making merits careful investigation [77]. Importantly, the open state of the field presents an opportunity to co-develop free text items for describing functional status in PROs alongside the NLP technologies used to analyze them. Entering at the ground level in designing both instrument items and analytic technologies provides an opportunity to ground the entire design and evaluation process in principles of equity, and to ensure the capacity to report the broadest set of contextual and patient factors affecting the experience of function. Doing so will directly support the development of patient-centered goals that reflect the unique lived experience and diverse needs of each person.

### A vision forward: Impacts of the proposed digital health directions in practice

The research and development directions we have proposed will integrate naturally with other technologies and approaches in the growing digital health ecosystem to support more successful and patient-centered care. Structured coding systems such as the ICD classifications or CPT have transformed automated clinical decision support systems and data-driven quality assessment; NLP-driven alignment of clinical observations with the ICF can integrate functional outcomes and facilitators into these processes as well [63]. Indexing information on function and context in EHR can not only support clinician-directed chart review to understand functional trajectories over time, but also enable automated visualization of functional measurements and support remote monitoring in patient care. Further, retrieval and display of patient experiences and priorities alongside clinical measures of function can help guide patient-centered interventions and interactions to improve health outcomes [78].

Our discussion focused specifically on clinical encounters, which are only one source of information about a person’s experience of function. While further work is needed to investigate barriers and facilitators in combining clinical data with other sources of person-centered information, there are clear implications of digital health tools for addressing disparities experienced by people with disabilities outside the clinic as well. Technologies drawing on EHR data can be combined with patient-generated health data from consumer devices to combine quantitative biological and biomechanical measures with clinical and patient-reported observations for a more holistic understanding of functional experience. Analysis of individual-level data can be used to identify persistent barriers to function across specific patient populations, informing changes in policy and practice aimed at reducing outcome disparities. Better information on function and its contributing factors can also inform financial decisions such as reimbursement for care or government disability benefits, which directly affect long-term functional outcomes, to reduce observed disparities by better addressing the specific needs and values of individuals [79,80]. However, as we discuss in the following section, these significant opportunities for positive impact can only be achieved through careful design and with a thorough awareness of the potential risks of inappropriately designed technologies.

### Limitations and the need for situated design

Our discussion thus far has focused on the potential for using digital health technologies as a tool for positive change in disabled people’s health equity. There are also important factors in the design, development, and management of these technologies that can serve as barriers to this positive change, and lead technologies (and technology-assisted processes) designed with good intentions to actually contribute to widening inequities. Achieving our vision in practice thus also requires both an awareness and careful navigation of these risks throughout the technology lifespan, from initial conceptualization to management and decommissioning of deployed technologies. We briefly discuss key risks here and emphasize the importance of further research and development of best practices around equitable and inclusive use of health information technologies, particularly when related to disability.

#### Access and accessibility

Inequities in access to healthcare services are widely recognized as major contributors to inequitable health outcomes. Access issues are exacerbated for disabled people and people with chronic health conditions, who may need access to highly specialized care that is often least available in the areas where it is needed most [81]. Adding digital health technologies into the picture can further compound access challenges: Technologies for data collection (e.g., mHealth, environmental sensors) are frequently expensive, difficult to source, and may not be covered by health insurance plans; while high-performance data analytics technologies (such as many NLP systems) may require expensive hardware or cloud computing services that are only realistically accessible to large medical centers [82,83]. Reliance on digital health technologies must therefore be balanced in each situation with careful consideration of economic, geographic, and cultural access of a patient population, so as not to further widen existing inequities. There is also a significant need for further research on reducing the cost of digital health technologies, for patients, providers, and health systems.

The usability and accessibility of digital health technologies is also a critical deciding factor in whether those technologies help or harm. While many of the uses of digital health discussed in this article are not patient-facing technologies but rather information-processing technologies without a direct user interface, there remain significant concerns around transparency and the role of the technology that must be addressed. The use of AI technologies within decision-making is often invisible to those who are affected by it (e.g., patients affected by AI analysis of their health data), and even visible technology use may still be opaque and difficult to explain [84]. In technologies that are user-facing, safety issues in both information display and user interaction have already been identified in consumer health apps [85]; the expansion of patient-facing tools for data collection, such as our proposal of collecting narratives of lived disability experience, presents further risks of inaccessible system design excluding those who are most meant to be included. Turning digital health directions into concrete technologies thus must include both accessibility of interaction and accessibility of understanding what the technology is doing and how.

#### Data and representation

The counterpart of ensuring that technologies are accessible is ensuring that the information recorded and analyzed with those technologies is both accurate and representative. Documentation errors are common [86], and time demands and burnout can reduce healthcare providers’ engagement with care and increase rates of medical errors [87], further affecting the quality of documentation (and therefore, the quality of any analysis of that documentation). Personal prejudices and biases may similarly affect the accuracy of documentation and the language used in it [88,89], and providers may lack the vocabulary or expertise to appropriately represent the experiences of marginalized patients [90]. Further, a lack of access to providers with appropriate specialized training may mean that even when patients with complex health needs do receive care, that care may not accurately reflect their needs and experiences. Digital health technologies, and particularly NLP technologies working directly with provider language, must therefore be designed with a sensitivity to the fidelity and the representativeness of the data collected in a given setting and ideally combined with multiple distinct data sources (including PRO tools) to provide as complete a picture as possible of the patient’s situation.

An important possibility for providing additional data sources, and directly incorporating the patient’s perspective into the EHR, lies in recording and transcribing healthcare encounters using speech to text technologies, thus making the patient–provider conversation itself available for NLP analysis. Automated transcription has shown significant promise in improving providers’ data entry workflows, and some preliminary studies have explored its use in analyzing clinical conversations directly [91]. These tools can provide direct access and opportunities for insight into the patient’s description of their own experience and priorities, in addition to what PRO tools elicit. However, conversational data imposes additional requirements on analytic pipelines, including accounting for the wide-ranging and continuous nature of a conversation (in contrast to discrete items in a survey or semi-structured provider notes) and the mixed registers of patient and provider language and information. Further research is needed to understand the challenges and opportunities of automated transcription, particularly in healthcare for disabled people.

#### Technology management and responsible use of machine learning

The implementation and management of digital health technologies poses a variety of further challenges, from logistical and economic as well as equity standpoints. Implementing digital health technologies in practice often requires complex integrations of health IT technologies and standards, as well as driving behavioral change in adopting and using the new technologies. Information about functional status and context in particular spreads across a disconnected constellation of IT systems in outpatient, inpatient, and transitional care and may be recorded in specialized systems within each setting. Patient-generated health data present its own challenges of data integration and communication between healthcare and consumer IT systems. Further, as measures of function and context continuously change and develop, it is not always clear what the most important or informative constructs are to capture in EHR and other health IT systems. It is not necessary to have complete solutions to these barriers before working on function-focused digital health technologies; rather, stakeholder engagement and feedback throughout the initial technology design process will help to clarify these issues in practice and lay out concrete, responsive steps to address them.

It is also important to actively engage with the risks and limitations of designing and building digital health technologies in practice, particularly where machine learning is involved. One key point of risk that must be taken into consideration is in the use of *pretrained* models as components of complex, application-oriented systems. Pretrained components are machine learning models that have been trained using separate data not specific to the task at hand, which are then used “off the shelf” either as fixed models within a larger system or as starting points for further machine learning on task-specific data. Pretrained language models are often used in NLP systems to represent the words and phrases analyzed by task-specific models, but these language models can reflect undesirable social biases (e.g., gender bias, racism, ableism) that may then inform the operation of the broader NLP system [92–95]. Nor are these issues restricted to NLP technologies, affecting speech recognition, computer vision, and others [96,97]. Beyond the use of pretrained components, the underlying data being analyzed in task-specific machine learning processes may exhibit demographic differences that are important to account for [98] or by similar token may reflect inequitable data collection practices [99]. In the pipelined technologies typically needed in practice, bias or errors early in the analytic process can continue to propagate through later stages of analysis and presentation [100]. Digital health technologies like those we propose must therefore be designed and evaluated through an intersectional lens to understand and mitigate the impact of demographic or conceptual biases throughout the analytic process, from data collection to algorithm design and beyond.

## Conclusions

People with disabilities experience significant health and healthcare disparities, which are further magnified when disability intersects with other marginalized identities. Addressing these disparities requires understanding both where they come from in an individual person’s experience and how they are reflected in differences in each person’s experience of function. EHR data, combined with self-reported information, provide an invaluable source for developing a multidimensional picture of individual functioning that can help identify broader actionable patterns underlying disparities. We identified 3 significant barriers to using EHR data to better understand the individual experience of function: challenges in documenting contextual factors of functioning, underemphasis of the patient’s perspective in health records, and the implementation gap for documenting functioning in current EHR systems. We described 3 directions for future research on digital health approaches to mitigate these barriers, such as combining the flexibility of free text documentation with NLP analytics to convert observations in text into actionable data. While our discussion focused on the clinical setting, the opportunities and the impacts for digital health and NLP research on function, context, and patient experience span across the entire lived experience of health. The directions outlined in this article provide a roadmap for advancing digital health research for disability and functional experience, with the expectation that specific directions, information needs, and decision-making priorities will be refined and grow together with our understanding over time. Digital health approaches drawing on the flexibility of NLP present a compelling opportunity to advance more equitable and person-centered decision-making processes in healthcare for people with disabilities.

## References

[1] Cieza A, Causey K, Kamenov K, Hanson SW, Chatterji S, Vos T. Global estimates of the need for rehabilitation based on the Global Burden of Disease study. A systematic analysis for the Global Burden of Disease Study 2019. Lancet. 2019;2020. doi: 10.1016/S0140-6736(20)32340-0 33275908 PMC7811204

[2] Meddings J, Reichert H, Smith SN, Iwashyna TJ, Langa KM, Hofer TP, et al. The Impact of Disability and Social Determinants of Health on Condition-Specific Readmissions beyond Medicare Risk Adjustments: A Cohort Study. J Gen Intern Med. 2017;32:71–80. doi: 10.1007/s11606-016-3869-x 27848189 PMC5215164

[3] Majer IM, Nusselder WJ, Mackenbach JP, Klijs B, van Baal PHM. Mortality risk associated with disability: a population-based record linkage study. Am J Public Health. 2011/10/20. 2011;101: e9–e15. doi: 10.2105/AJPH.2011.300361 22021307 PMC3222426

[4] Vogeli C, Shields AE, Lee TA, Gibson TB, Marder WD, Weiss KB, et al. Multiple Chronic Conditions: Prevalence, Health Consequences, and Implications for Quality, Care Management, and Costs. J Gen Intern Med. 2007;22:391–395. doi: 10.1007/s11606-007-0322-1 18026807 PMC2150598

[5] Iezzoni LI. Eliminating Health And Health Care Disparities Among The Growing Population Of People With Disabilities. Health Affairs. 2011;30:1947–1954. doi: 10.1377/hlthaff.2011.0613 21976339

[6] Cheak-Zamora NC, Thullen M. Disparities in Quality and Access to Care for Children with Developmental Disabilities and Multiple Health Conditions. Matern Child Health J. 2017;21:36–44. doi: 10.1007/s10995-016-2091-0 27423238

[7] Krahn GL, Walker DK, Correa-De-Araujo R. Persons With Disabilities as an Unrecognized Health Disparity Population. Am J Public Health. 2015;105:S198–S206. doi: 10.2105/AJPH.2014.302182 25689212 PMC4355692

[8] Meade MA, Mahmoudi E, Lee S-Y. The intersection of disability and healthcare disparities: a conceptual framework. Disabil Rehabil. 2015;37:632–641. doi: 10.3109/09638288.2014.938176 25060038

[9] World Health Organization. International Classification of Functioning, Disability and Health: ICF. Geneva: World Health Organization; 2001.

[10] Maroto M, Pettinicchio D, Patterson AC. Hierarchies of Categorical Disadvantage: Economic Insecurity at the Intersection of Disability, Gender, and Race. Gend Soc. 2018;33:64–93. doi: 10.1177/0891243218794648

[11] Banks J. Invisible man: examining the intersectionality of disability, race, and gender in an urban community. Disabil Soc. 2018;33:894–908. doi: 10.1080/09687599.2018.1456912

[12] Chapman EN, Kaatz A, Carnes M. Physicians and Implicit Bias: How Doctors May Unwittingly Perpetuate Health Care Disparities. J Gen Intern Med. 2013;28:1504–1510. doi: 10.1007/s11606-013-2441-1 23576243 PMC3797360

[13] Fuentes MM, Moore M, Qiu Q, Quistberg A, Frank M, Vavilala MS. Differences in Injury Characteristics and Outcomes for American Indian/Alaska Native People Hospitalized with Traumatic Injuries: an Analysis of the National Trauma Data Bank. J Racial Ethn Heal Disparities. 2019;6:335–344. doi: 10.1007/s40615-018-0529-3 30276637 PMC6424619

[14] Hurwitz M, Fuentes M. Healthcare Disparities in Dysvascular Lower Extremity Amputations. Curr Phys Med Rehabil Reports. 2020;8:110–117. doi: 10.1007/s40141-020-00281-5

[15] Resnik L, Borgia M. Predicting prosthetic prescription after major lower-limb amputation. J Rehabil Res Dev. 2015;52:641–652. doi: 10.1682/JRRD.2014.09.0216 26562228

[16] Freburger JK, Holmes GM, Ku L-JE, Cutchin MP, Heatwole-Shank K, Edwards LJ. Disparities in postacute rehabilitation care for stroke: an analysis of the state inpatient databases. Arch Phys Med Rehabil. 2011;92:1220–1229. doi: 10.1016/j.apmr.2011.03.019 21807141 PMC4332528

[17] Asemota AO, George BP, Cumpsty-Fowler CJ, Haider AH, Schneider EB. Race and insurance disparities in discharge to rehabilitation for patients with traumatic brain injury. J Neurotrauma. 2013;30:2057–2065. doi: 10.1089/neu.2013.3091 23972035 PMC3868359

[18] Mitra S, Palmer M, Kim H, Mont D, Groce N. Extra costs of living with a disability: A review and agenda for research. Disabil Health J. 2017;10:475–484. doi: 10.1016/j.dhjo.2017.04.007 28501322

[19] Disability Emmett T., poverty, gender and race. In: Watermeyer B, Swartz L, Lorenzo T, Schneider M, Priestley M, editors. Disability and Social Change: A South African Agenda. Cape Town, South Africa: HSRC Press; 2006. p. 207–233.

[20] Brown AF, Ma GX, Miranda J, Eng E, Castille D, Brockie T, et al. Structural Interventions to Reduce and Eliminate Health Disparities. Am J Public Health. 2019;109:S72–S78. doi: 10.2105/AJPH.2018.304844 30699019 PMC6356131

[21] Bettger JP, Nguyen VQC, Thomas JG, Guerrier T, Yang Q, Hirsch MA, et al. Turning Data Into Information: Opportunities to Advance Rehabilitation Quality, Research, and Policy. Arch Phys Med Rehabil. 2018/01/31. 2018;99: 1226–1231. doi: 10.1016/j.apmr.2017.12.029 29407515 PMC6571032

[22] Nicosia FM, Spar MJ, Steinman MA, Lee SJ, Brown RT. Making Function Part of the Conversation: Clinician Perspectives on Measuring Functional Status in Primary Care. J Am Geriatr Soc. 2019;67:493–502. doi: 10.1111/jgs.15677 30506667 PMC6402957

[23] Andrews EE, Forber-Pratt AJ, Mona LR, Lund EM, Pilarski CR, Balter R. #SaytheWord: A disability culture commentary on the erasure of “disability”. Rehabilitation Psychology. Andrews, Erin E.: Central Texas Veterans Health Care System, Austin VA Outpatient Clinic, Mental Health & Behavioral Medicine Service (116B), 7901 Metropolis Drive, Austin, TX, US, 78744, erin.andrews2@va.gov: American Psychological Association; 2019. p. 111–118. doi: 10.1037/rep0000258 30762412

[24] Dunn DS, Andrews EE. Person-first and identity-first language: Developing psychologists’ cultural competence using disability language. Am Psychol. 2015;70:255–264. doi: 10.1037/a0038636 25642702

[25] Haley SM, Coster WJ, Binda-Sundberg K. Measuring Physical Disablement: The Contextual Challenge. Phys Ther. 1994;74:443–451. doi: 10.1093/ptj/74.5.443 8171106

[26] Sinclair CM, Meredith P, Strong J, Feeney R. Personal and Contextual Factors Affecting the Functional Ability of Children and Adolescents with Chronic Pain: A Systematic Review. J Dev Behav Pediatr. 2016;37. Available from: doi: 10.1097/DBP.0000000000000300 27096569

[27] Stolwijk C, Castillo-Ortiz J-D, Gignac M, Luime J, Boonen A, Group for the OWP. Importance of Contextual Factors When Measuring Work Outcome in Ankylosing Spondylitis: A Systematic Review by the OMERACT Worker Productivity Group. Arthritis Care Res (Hoboken). 2015;67:1316–1327. doi: 10.1002/acr.22573 25732705

[28] Whiteneck G, Dijkers MP. Difficult to Measure Constructs: Conceptual and Methodological Issues Concerning Participation and Environmental Factors. Arch Phys Med Rehabil. 2009;90:S22–S35. doi: 10.1016/j.apmr.2009.06.009 19892071

[29] Jette AM, Ni P, Rasch E, Marfeo E, McDonough C, Brandt D, et al. The Work Disability Functional Assessment Battery (WD-FAB). Phys Med Rehabil Clin. 2019;30:561–572. doi: 10.1016/j.pmr.2019.03.004 31227131 PMC10123957

[30] Nussbaum R, Kelly C, Quinby E, Mac A, Parmanto B, Dicianno BE. Systematic Review of Mobile Health Applications in Rehabilitation. Arch Phys Med Rehabil. 2019;100:115–127. doi: 10.1016/j.apmr.2018.07.439 30171827

[31] Lucyk K, McLaren L. Taking stock of the social determinants of health: A scoping review. PLoS ONE. 2017;12:e0177306. doi: 10.1371/journal.pone.0177306 28493934 PMC5426664

[32] Feller DJ, Bear Don’t Walk IV OJ, Zucker, Yin MT, Gordon P, Elhadad N. Detecting Social and Behavioral Determinants of Health with Structured and Free-Text Clinical Data. Appl Clin Inf. 2020;11:172–181. doi: 10.1055/s-0040-1702214 32131117 PMC7056402

[33] Cantor MN, Thorpe L. Integrating Data On Social Determinants Of Health Into Electronic Health Records. Health Affairs. 2018;37:585–590. doi: 10.1377/hlthaff.2017.1252 29608369 PMC10995852

[34] Friedman C, Owen AL. Defining disability: Understandings of and attitudes towards ableism and disability. Disabil Stud Q. 2017;37.

[35] Shakespeare T, Iezzoni LI, Groce NE. Disability and the training of health professionals. Lancet. 2009;374:1815–1816. doi: 10.1016/s0140-6736(09)62050-x 19957403

[36] Carel H, Kidd IJ. Epistemic injustice in healthcare: a philosophial analysis. Med Heal Care Philos. 2014;17:529–540. doi: 10.1007/s11019-014-9560-2 24740808

[37] Ho A. Trusting experts and epistemic humility in disability. IJFAB Int J Fem Approaches to Bioeth. 2011;4:102–123. doi: 10.3138/ijfab.4.2.102

[38] Peña-Guzmán DM, Reynolds JM. The harm of ableism: Medical error and epistemic injustice. Kennedy Inst Ethics J. 2019;29:205–242. doi: 10.1353/ken.2019.0023 31656232

[39] Jones M. Deafness as culture: A psychosocial perspective. Disabil Stud Q. 2002:22.

[40] Smith DL. Disparities in patient-physician communication for persons with a disability from the 2006 Medical Expenditure Panel Survey (MEPS). Disabil Health J. 2009;2:206–215. doi: 10.1016/j.dhjo.2009.06.002 21122761

[41] Pieterse AH, Finset A. Shared decision making—Much studied, much still unknown. Patient Educ Couns. 2019;102:1946–1948. doi: 10.1016/j.pec.2019.09.006 31582048

[42] Hoffmann TC, Lewis J, Maher CG. Shared decision making should be an integral part of physiotherapy practice. Physiotherapy. 2020;107:43–49. doi: 10.1016/j.physio.2019.08.012 32026834

[43] Rosenbloom ST, Denny JC, Xu H, Lorenzi N, Stead WW, Johnson KB. Data from clinical notes: a perspective on the tension between structure and flexible documentation. J Am Med Inform Assoc. 2011;18:181–186. doi: 10.1136/jamia.2010.007237 21233086 PMC3116264

[44] Vreeman DJ, Richoz C. Possibilities and implications of using the ICF and other vocabulary standards in electronic health records. Physiother Res Int. 2015;20:210–219. doi: 10.1002/pri.1559 23897840 PMC3907616

[45] Chesbrough K, Elrod M, Irrgang JJ. Systems Science in Rehabilitation Practice Realized. Phys Ther. 2018;98:909–910. doi: 10.1093/ptj/pzy093 30101311

[46] Maritz R, Baptiste S, Darzins SW, Magasi S, Weleschuk C, Prodinger B. Linking occupational therapy models and assessments to the ICF to enable standardized documentation of functioning. Can J Occup Ther. 2018;85:330–341. doi: 10.1177/0008417418797146 30442023

[47] Mayo NE, Poissant L, Ahmed S, Finch L, Higgins J, Salbach NM, et al. Incorporating the International Classification of Functioning, Disability, and Health (ICF) into an Electronic Health Record to Create Indicators of Function: Proof of Concept Using the SF-12. J Am Med Informatics Assoc. 2004;11:514–522. Available from: 10.1197/jamia.M1462. PMC524632 15298994

[48] Mahmoud R, El-Bendary N, Mokhtar HMO, Hassanien AE. ICF based automation system for spinal cord injuries rehabilitation. 2014 9th International Conference on Computer Engineering Systems (ICCES). Cairo, Egypt; 2014. p. 192–197. doi: 10.1109/ICCES.2014.7030955

[49] Bogardus ST, Towle V, Williams CS, Desai MM, Inouye S. What Does the Medical Record Reveal about Functional Status? J Gen Intern Med. 2001;16:728–736. doi: 10.1111/j.1525-1497.2001.00625.x 11722685 PMC1495285

[50] Agaronnik N, Lindvall C, El-Jawahri A, He W, Iezzoni L. Use of Natural Language Processing to Assess Frequency of Functional Status Documentation for Patients Newly Diagnosed With Colorectal Cancer. JAMA Oncol. 2020;6:1628–1630. doi: 10.1001/jamaoncol.2020.2708 32880603 PMC7489406

[51] Gesner E, Gazarian P, Dykes P. The Burden and Burnout in Documenting Patient Care: An Integrative Literature Review. Stud Health Technol Inform. 2019;264:1194–1198. doi: 10.3233/SHTI190415 31438114

[52] Stucki G, Bickenbach J. Functioning information in the learning health system. Eur J Phys Rehabil Med. 2017;53:139–143. doi: 10.23736/S1973-9087.17.04612-3 28145399

[53] Newman-Griffis D, Porcino J, Zirikly A, Thieu T, Camacho Maldonado J, Ho P-S, et al. Broadening horizons: the case for capturing function and the role of health informatics in its use. BMC Public Health. 2019;19:1288. doi: 10.1186/s12889-019-7630-3 31615472 PMC6794808

[54] Gustavsen M, Mengshoel AM. Clinical physiotherapy documentation in stroke rehabilitation: an ICIDH-2 beta-2 based analysis. Disabil Rehabil. 2003;25:1089–1096. doi: 10.1080/0963828031000148629 12944148

[55] Skube SJ, Lindemann EA, Arsoniadis EG, Akre M, Wick EC, Melton GB. Characterizing Functional Health Status of Surgical Patients in Clinical Notes. AMIA Joint Summits on Translational Science Proceedings 2018. San Francisco, California, USA: American Medical Informatics Association; 2018. p. 379–388. PMC5961772 29888096

[56] Kuang J, Mohanty AF, Rashmi VH, Weir CR, Bray BE, Zeng-Treitler Q. Representation of Functional Status Concepts from Clinical Documents and Social Media Sources by Standard Terminologies. AMIA Annual Symposium Proceedings 2015. San Francisco, California, USA: American Medical Informatics Association; 2015. p. 795–803. Available from: http://www.ncbi.nlm.nih.gov/pmc/articles/PMC4765559/. PMC4765559 26958215

[57] Ruggieri AP, Pakhomov SV, Chute CG. A corpus driven approach applying the “frame semantic” method for modeling functional status terminology. Stud Health Technol Inform. 2004;107:434–438. doi: 10.3233/978-1-60750-949-3-434 15360850

[58] Thieu T, Maldonado JC, Ho P-S, Ding M, Marr A, Brandt D, et al. A comprehensive study of mobility functioning information in clinical notes: Entity hierarchy, corpus annotation, and sequence labeling. Int J Med Inform. 2021;147:104351. doi: 10.1016/j.ijmedinf.2020.104351 33401169 PMC8104034

[59] Kukafka R, Bales ME, Burkhardt A, Friedman C. Human and Automated Coding of Rehabilitation Discharge Summaries According to the International Classification of Functioning, Disability, and Health. J Am Med Informatics Assoc. 2006;13:508–515. doi: 10.1197/jamia.M2107 16799117 PMC1561799

[60] Agaronnik ND, Lindvall C, El-Jawahri A, He W, Iezzoni LI. Challenges of Developing a Natural Language Processing Method With Electronic Health Records to Identify Persons With Chronic Mobility Disability. Arch Phys Med Rehabil. 2020;101:1739–1746. doi: 10.1016/j.apmr.2020.04.024 32446905 PMC7529728

[61] Newman-Griffis D, Fosler-Lussier E. HARE: a Flexible Highlighting Annotator for Ranking and Exploration. Proceedings of the 2019 Conference on Empirical Methods in Natural Language Processing and the 9th International Joint Conference on Natural Language Processing (EMNLP-IJCNLP): System Demonstrations. Hong Kong, China: Association for Computational Linguistics; 2019. p. 85–90. doi: 10.18653/v1/D19-3015 PMC7731636 33313604

[62] Newman-Griffis D, Fosler-Lussier E. Automated Coding of Under-Studied Medical Concept Domains: Linking Physical Activity Reports to the International Classification of Functioning, Disability, and Health. Front Digit Heal. 2021;3:620828. doi: 10.3389/fdgth.2021.620828 33791684 PMC8009547

[63] Newman-Griffis D, Camacho Maldonado J, Ho P-S, Sacco M, Jimenez Silva R, Porcino J, et al. Linking Free Text Documentation of Functioning and Disability to the ICF with Natural Language Processing. Front Rehabil Sci. 2021;2:742702. doi: 10.3389/fresc.2021.742702 35694445 PMC9180751

[64] Kharrazi H, Anzaldi LJ, Hernandez L, Davison A, Boyd CM, Leff B, et al. The Value of Unstructured Electronic Health Record Data in Geriatric Syndrome Case Identification. J Am Geriatr Soc. 2018;66:1499–1507. doi: 10.1111/jgs.15411 29972595

[65] Chen T, Dredze M, Weiner JP, Kharrazi H. Identifying vulnerable older adult populations by contextualizing geriatric syndrome information in clinical notes of electronic health records. J Am Med Informatics Assoc. 2019;26:787–795. doi: 10.1093/jamia/ocz093 31265063 PMC7647225

[66] Shao Y, Mohanty AF, Ahmed A, Weir CR, Bray BE, Shah RU, et al. Identification and Use of Frailty Indicators from Text to Examine Associations with Clinical Outcomes Among Patients with Heart Failure. AMIA Annual Symposium Proceedings. American Medical Informatics Association; 2016. p. 1110–1118. Available from: https://www.ncbi.nlm.nih.gov/pubmed/28269908. PMC5333331 28269908

[67] Fougeyrollas P. Documenting environmental factors for preventing the handicap creation process: Quebec contributions relating to ICIDH and social participation of people with functional differences. Disabil Rehabil. 1995;17:145–153. doi: 10.3109/09638289509166709 7787197

[68] Gomez SL, Shariff-Marco S, DeRouen M, Keegan THM, Yen IH, Mujahid M, et al. The impact of neighborhood social and built environment factors across the cancer continuum: Current research, methodological considerations, and future directions. Cancer. 2015;121:2314–2330. doi: 10.1002/cncr.29345 25847484 PMC4490083

[69] Simeonsson RJ, Lollar D, Björck-Åkesson E, Granlund M, Brown SC, Zhuoying Q, et al. ICF and ICF-CY lessons learned: Pandora’s box of personal factors. Disabil Rehabil. 2014;36:2187–2194. doi: 10.3109/09638288.2014.892638 24601863

[70] Basch E, Barbera L, Kerrigan CL, Velikova G. Implementation of Patient-Reported Outcomes in Routine Medical Care. Am Soc Clin Oncol Educ B. 2018:122–134. doi: 10.1200/EDBK_200383 30231381

[71] Bottomley A, Reijneveld JC, Koller M, Flechtner H, Tomaszewski KA, Greimel E, et al. Current state of quality of life and patient-reported outcomes research. Eur J Cancer. 2019;121:55–63. doi: 10.1016/j.ejca.2019.08.016 31561134

[72] Phillips L, Carsen S, Vasireddi A, Mulpuri K. Use of Patient-reported Outcome Measures in Pediatric Orthopaedic Literature. J Pediatr Orthop. 2018;38:393–397. doi: 10.1097/BPO.0000000000000847 27603185

[73] van Leeuwen LM, Pronk M, Merkus P, Goverts ST, Anema JR, Kramer SE. Barriers to and enablers of the implementation of an ICF-based intake tool in clinical otology and audiology practice—A qualitative pre-implementation study. PLoS ONE. 2018;13:e0208797. doi: 10.1371/journal.pone.0208797 30533057 PMC6289452

[74] Mathews M, Agniel D, Elliott MN, Martino SC, Guerino P, Orr N, et al. The development of a patient-reported functional limitations index. Am J Manag Care. 2020;26:e225–e231. doi: 10.37765/ajmc.2020.43765 32672921

[75] Gensheimer SG, Wu AW, Snyder CF. Oh, the Places We’ll Go: Patient-Reported Outcomes and Electronic Health Records. Patient. 2018:591–598. doi: 10.1007/s40271-018-0321-9 29968179

[76] Walker J, Leveille S, Bell S, Chimowitz H, Dong Z, Elmore JG, et al. OpenNotes After 7 Years: Patient Experiences With Ongoing Access to Their Clinicians’ Outpatient Visit Notes. J Med Internet Res. 2019;21:e13876. doi: 10.2196/13876 31066717 PMC6526690

[77] Brédart A, Marrel A, Abetz-Webb L, Lasch K, Acquadro C. Interviewing to develop Patient-Reported Outcome (PRO) measures for clinical research: eliciting patients’ experience. Health Qual Life Outcomes. 2014;12:15. doi: 10.1186/1477-7525-12-15 24499454 PMC3933509

[78] Thornton RLJ, Glover CM, Cené CW, Glik DC, Henderson JA, Williams DR. Evaluating Strategies For Reducing Health Disparities By Addressing The Social Determinants Of Health. Health Aff (Millwood). 2016;35:1416–1423. doi: 10.1377/hlthaff.2015.1357 27503966 PMC5524193

[79] Grobaty L, Lajam C, Hutzler L. Impact of Value-Based Reimbursement on Health-Care Disparities for Total Joint Arthroplasty Candidates. JBJS Rev. 2020;8:e2000073. doi: 10.2106/JBJS.RVW.20.00073 33186211

[80] Godtland EM, Grgich M, Petersen CD, Sloane DM, Walker ANNT. Racial Disparities In Federal Disability Benefits. Contemp Econ Policy. 2007;25:27–45. 10.1111/j.1465-7287.2006.00031.x

[81] Gulley SP, Rasch EK, Bethell CD, Carle AC, Druss BG, Houtrow AJ, et al. At the intersection of chronic disease, disability and health services research: A scoping literature review. Disabil Health J. 2018;11:192–203. doi: 10.1016/j.dhjo.2017.12.012 29396271 PMC5869152

[82] Yao R, Zhang W, Evans R, Cao G, Rui T, Shen L. Inequities in Health Care Services Caused by the Adoption of Digital Health Technologies: Scoping Review. J Med Int Res. 2022;24:e34144. doi: 10.2196/34144 35311682 PMC8981004

[83] Panch T, Mattie H, Celi LA. The “inconvenient truth” about AI in healthcare. NPJ Digit Med. 2019;2:77. doi: 10.1038/s41746-019-0155-4 31453372 PMC6697674

[84] Ehsan U, Liao QV, Muller M, Riedl MO, Weisz JD. Expanding Explainability: Towards Social Transparency in AI Systems. Proceedings of the 2021 CHI Conference on Human Factors in Computing Systems. New York, NY, USA: Association for Computing Machinery; 2021. doi: 10.1145/3411764.3445188

[85] Akbar S, Enrico C, Magrabi F. Safety concerns with consumer-facing mobile health applications and their consequences: a scoping review. J Am Med Informatics Assoc. 2020;27:330–340. doi: 10.1093/jamia/ocz175 31599936 PMC7025360

[86] Weiner SJ, Wang S, Kelly B, Sharma G, Schwartz A. How accurate is the medical record? A comparison of the physician’s note with a concealed audio recording in unannounced standardized patient encounters. J Am Med Inform Assoc. 2020;27:770–775. doi: 10.1093/jamia/ocaa027 32330258 PMC7647276

[87] Dyrbye L, Shanafelt T. A narrative review on burnout experienced by medical students and residents. Med Educ. 2016;50:132–149. doi: 10.1111/medu.12927 26695473

[88] Beach MC, Saha S, Park J, Taylor J, Drew P, Plank E, et al. Testimonial Injustice: Linguistic Bias in the Medical Records of Black Patients and Women. J Gen Intern Med. 2021;36:1708–1714. doi: 10.1007/s11606-021-06682-z 33754318 PMC8175470

[89] Sun M, Oliwa T, Peek ME, Tung EL. Negative Patient Descriptors: Documenting Racial Bias In The Electronic Health Record. Health Affairs. 2022;41:203–211. doi: 10.1377/hlthaff.2021.01423 35044842 PMC8973827

[90] Kronk CA, Everhart AR, Ashley F, Thompson HM, Schall TE, Goetz TG, et al. Transgender data collection in the electronic health record: Current concepts and issues. J Am Med Informatics Assoc. 2022;29:271–284. doi: 10.1093/jamia/ocab136 34486655 PMC8757312

[91] van Buchem MM, Boosman H, Bauer MP, Kant IMJ, Cammel SA, Steyerberg EW. The digital scribe in clinical practice: a scoping review and research agenda. NPJ Digit Med. 2021;4:57. doi: 10.1038/s41746-021-00432-5 33772070 PMC7997964

[92] Garg N, Schiebinger L, Jurafsky D, Zou J. Word embeddings quantify 100 years of gender and ethnic stereotypes. Proc Natl Acad Sci U S A. 2018;115:E3635–E3644. doi: 10.1073/pnas.1720347115 29615513 PMC5910851

[93] Zhao J, Wang T, Yatskar M, Cotterell R, Ordonez V, Chang K-W. Gender Bias in Contextualized Word Embeddings. Proceedings of the 2019 Conference of the North {A}merican Chapter of the Association for Computational Linguistics: Human Language Technologies, Volume 1 (Long and Short Papers). Minneapolis, Minnesota: Association for Computational Linguistics; 2019. p. 629–634. doi: 10.18653/v1/N19-1064

[94] Hutchinson B, Prabhakaran V, Denton E, Webster K, Zhong Y, Denuyl S. Social Biases in NLP Models as Barriers for Persons with Disabilities. Proceedings of the 58th Annual Meeting of the Association for Computational Linguistics. Online: Association for Computational Linguistics. 2020. p. 5491–5501. doi: 10.18653/v1/2020.acl-main.487

[95] Bolukbasi T, Chang K-W, Zou JY, Saligrama V, Kalai AT. Man is to computer programmer as woman is to homemaker? Debiasing word embeddings. Adv Neural Inf Process Syst. 2016;29.

[96] Koenecke A, Nam A, Lake E, Nudell J, Quartey M, Mengesha Z, et al. Racial disparities in automated speech recognition. Proc Natl Acad Sci U S A. 2020;117:7684–7689. doi: 10.1073/pnas.1915768117 32205437 PMC7149386

[97] Buolamwini JA. Gender shades: intersectional phenotypic and demographic evaluation of face datasets and gender classifiers. Massachusetts Institute of Technology. 2017.

[98] Lester D, Linn M. Sex differences in suicide notes. Psychol Rep. 1997;80:1302. doi: 10.2466/pr0.1997.80.3c.1302 9246894

[99] Gianfrancesco MA, Tamang S, Yazdany J, Schmajuk G. Potential Biases in Machine Learning Algorithms Using Electronic Health Record Data. JAMA Intern Med. 2018;178:1544–1547. doi: 10.1001/jamainternmed.2018.3763 30128552 PMC6347576

[100] Ferraro JP. Reducing pipeline error propagation in natural language processing: Part-of-speech tagging applied to clinical narratives. The University of Utah; 2013.

